# Overprediction of mortality with the Hunt and Hess score in aneurysmal subarachnoid hemorrhage: retrospective multicenter study

**DOI:** 10.62675/2965-2774.20260403

**Published:** 2026-06-03

**Authors:** Maria Victoria Gonzalez, Carlos Gustavo Videla, Melany Berdiñas Anfuso, Florencia Monsalve, Giuliano Yossa, Sol Prati, Maria Sofia Venuti, Sofía Schverdfinger, Alicia Roxana Gira, Vladimir Ortega, Daniel Ivulich, Ivan Alfredo Huespe, Nicolas Marcelo Ciarrocchi

**Affiliations:** 1 Hospital Italiano de Buenos Aires Buenos Aires Argentina Hospital Italiano de Buenos Aires - Buenos Aires, Argentina.; 2 Hospital Universitario Austral Buenos Aires Argentina Hospital Universitario Austral - Buenos Aires, Argentina.; 3 Hospital Alemán Buenos Aires Argentina Hospital Alemán - Buenos Aires, Argentina.

## INTRODUCTION

Aneurysmal subarachnoid hemorrhage (aSAH) accounts for 5% of strokes and is still associated with high morbidity and mortality, with case fatality approaching 50%.^(1)^ Clinical prediction models are essential for outcome estimation.^(2)^ The Hunt and Hess (HH) and World Federation of Neurosurgical Societies (WFNS) scales are widely used to determine severity and prognosis at admission.^(3,4)^ However, their predictive accuracy in contemporary neurocritical care is uncertain, as early aneurysm repair and improved intensive care unit (ICU) management have significantly modified survival patterns.^(5,6)^ This study evaluated the predictive performance of the HH score for predicting in-hospital mortality and described neurological outcomes at discharge and 6 months in a multicenter Latin American cohort.

## METHODS

A retrospective cohort study was performed from 2011 to 2022 across three high-complexity hospitals in Argentina (Hospital Alemán, Hospital Universitario Austral, and Hospital Italiano de Buenos Aires).

Adults (≥ 18 years) admitted to the ICU with angiographically confirmed non-traumatic aSAH were included. Patients with SAH secondary to neurosurgical manipulation or without aneurysmal confirmation were excluded. Inclusion occurred at ICU admission following aSAH diagnosis. Collected data included demographics, Acute Physiology and Chronic Health Evaluation II (APACHE II), HH grade, treatment modality, and neurological outcomes using the modified Rankin Scale (mRS) at discharge and 6 months. Discrimination was evaluated by the area under the Receiver Operating Characteristic curve (AUROC), and calibration was assessed by comparing predicted versus observed mortality and by Cox-based calibration statistics. Statistical analyses are detailed in the Supplementary Material.

## RESULTS

Of 222 screened patients, 175 met inclusion criteria (mean age 59 ± 14 years; mean APACHE II 13 ± 8.4). The most frequent HH grade was 2 (n = 64; 36.6%). Overall, in-hospital mortality was 21% (n = 37). The HH score showed acceptable discrimination for mortality prediction (AUROC 0.74; 95%CI 0.66 - 0.82) but poor calibration (calibration-in-the-large −3.6; slope 0.3), systematically overestimating mortality across all grades. Predicted mortality was 10% for HH grade 1 (observed 0%) and 99% for grade 5 (observed 46%).

**Figure 1:**
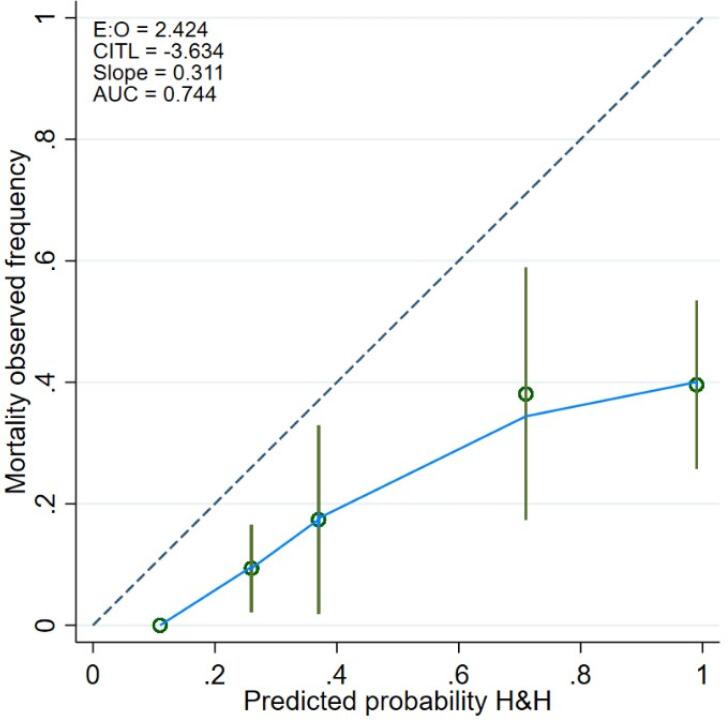
Calibration plot with the relation between expected and observed mortality probability.

Baseline characteristics and outcomes by HH grade are summarized in table 1. The calibration curve demonstrated overestimation across all categories (Figure 1). At discharge, 49% achieved mRS ≤ 3, increasing to 54% at 6 months. Among HH four and five patients, the favorable outcome increased from 9% to 22% at 6 months. Differences between ICU survivors and non-survivors, including functional outcomes and treatment strategies, are summarized in table 2. The 1-year and 5-year survival rates were 72% and 70%, respectively. Additional results are presented in the Supplementary Material.

**Table 1:** Demographic characteristics

Variable		Total of patients (n = 175)	Survivors at discharge (n = 138)	Deceased in ICU (n = 37)	p value
Age (years)		59.1 (14.4)	57.7 (14.6)	63.9 (13.0)	0.015
Sex		117 (66.9)	88 (63.8)	29 (78.4)	0.139
APACHE II		13.0 (8.47)	11.3 (8.01)	19.1 (7.30)	< 0.001
Charlson		2.00 [0.00 - 4.00]	1.00 [0.00 - 3.00]	3.00 [1.00 - 4.00]	0.029
Comorbidities					
	Smoking	41 (24.4)	32 (24.1)	9 (25.7)	0.99
	Diabetes	10 (5.71)	7 (5.07)	3 (8.11)	0.443
	Hypertension	75 (42.9)	59 (42.8)	16 (43.2)	0.99
	Chronic kidney disease	4 (2.30)	3 (2.19)	1 (2.70)	0.99
Need for mechanical ventilation		116 (66.3)	82 (59.4)	34 (91.9)	< 0.001
Days of mechanical ventilation		18.5 [5.25 - 28.8]	20.0 [6.75 - 29.0]	7.50 [3.00 - 16.0]	0.013
Hunt and Hess					< 0.001
	1	19 (10.9)	19 (13.8)	0 (0.00)	
	2	64 (36.6)	58 (42.0)	6 (16.2)	
	3	23 (13.1)	19 (13.8)	4 (10.8)	
	4	21 (12.0)	13 (9.42)	8 (21.6)	
	5	48 (27.)	29 (21.0)	19 (51.4)	
Fisher's modified scale					0.004
	1	8 (4.62)	8 (5.88)	0 (0.00)	
	2	17 (9.83)	17 (12.5)	0 (0.00)	
	3	39 (22.5)	34 (25.0)	5 (13.5)	
	4	109 (63.0)	77 (56.6)	32 (86.5)	
Hydrocephalus		69 (39.7)	45 (32.8)	24 (64.9)	0.001
Presence of neurological deficit		67 (38.5)	49 (35.5)	18 (50.0)	0.162

ICU - intensive care unit. Results expressed as mean (standard deviation), n (%), or median [interquartile range].

**Table 2:** Differences between intensive care unit survivors and non-survivors

Variable		All (n = 175)	ICU Survivors (n = 138)	Deceased in ICU (n = 37)	p value
Modified ranking at patient discharge					< 0.001
	mRS ≤ 3	85 (48.57)	85 (61,59)	0 (0)	
	mRs > 4	91 (52,0)	54 (39,13)	37 (100)	
Presence of vasospasm		60 (34.3)	51 (37.0)	9 (24.3)	0.214
Cerebrospinal fluid drainage		74 (42.3)	54 (39.1)	20 (54.1)	0.149
Aneurysm treatment					0.002
	No treatment is performed	13 (7.4)	8 (5.80)	5 (13.5)	
	Surgical	56 (32.0)	52 (37.7)	4 (10.8)	
	Endovascular	106 (60.6)	78 (56.5)	28 (75.7)	
Modified ranking 6 months after patient discharge					< 0.001
	mRS ≤ 3	76 (53,52)	76 (71,7)	0 (0)	
	mRS > 4	67 (46.48)	30 (28,3)	37 (100)	

ICU - intensive care unit; mRS - modified Rankin Scale. Results expressed as n (%).

## DISCUSSION

The HH score demonstrated good discrimination but poor calibration for in-hospital mortality prediction, substantially overestimating mortality, especially in high-grade cases. These findings align with recent international cohorts reporting acceptable discrimination but weak calibration. Nguyen et al. and Rojas-Panta et al. reported good discrimination of the HH scale (AUROC 0.84 - 0.71).^(7,8)^ However, calibration was not assessed in these studies, and predicted mortality differed from observed mortality despite good discrimination.

The overprediction likely reflects contemporary improvements in aneurysm repair and neurocritical care, which have reduced mortality even in severe cases.^(5,9)^ Comparison with WFNS and Glasgow coma scale (GCS) suggests that HH performs similarly in discrimination but tends to overestimate risk, particularly in poor neurological grades, consistent with prior studies.^(7)^ Patient-level factors, including age, sex, and APACHE II, not captured by the HH score, may partly explain the poor calibration observed. However, formal meta-regression was not feasible due to sample size limitations. Neurological recovery, particularly among patients with HH grades 4 - 5, continues beyond ICU discharge, supporting cautious prognostication and avoiding early withdrawal-of-care decisions.^(10)^

This study is one of the first multicenter prognostic evaluations of aSAH in Latin America. Limitations include its retrospective design, limited follow-up, and modest sample size.

In conclusion, although widely used, the HH score overpredicts mortality in contemporary aSAH care and should not be used as the sole parameter for clinical decision-making.

## Data Availability

Data is available on demand from referees.
