# Supplementary material for: Overprediction of mortality with the Hunt and Hess score in aneurysmal subarachnoid hemorrhage: retrospective multicenter study
Source: Crit Care Sci. 2026 Jun 3;38:e20260403. doi: 10.62675/2965-2774.20260403 (PMC13399240; doi:10.62675/2965-2774.20260403)
Supplement: Supplementary Material [file 2965-2774-ccsci-38-e20260403-suppl01.pdf]

Critical Care Science
https://doi.org/10.62675/2965-2774.20260403

SUPPLEMENTARY MATERIAL

Overprediction of mortality with the Hunt and Hess score in aneurysmal subarachnoid hemorrhage: retrospective multicenter study

Maria Victoria Gonzalez[1], Carlos Gustavo Videla[1], Melany Berdiñas Anfuso[1], Florencia Monsalve[2], Giuliano Yossa[3], Sol Prati[1], Maria Sofia Venuti[1], Sofía Schverdfinger[1], Alicia Roxana Gira[2], Vladimir Ortega[3], Daniel Ivulich[3], Ivan Alfredo Huespe[1], Nicolas Marcelo Ciarrocchi[1]

DESIGN AND SETTING

The approval of this study was obtained from the Institutional Review Board of all centers (protocol 5530). All data were obtained from electronic medical records. Informed Consent was waived due to the study's retrospective nature, the use of de-identified data, and the minimal risk to participants.

INTENSIVE CARE UNIT CHARACTERISTICS

All participating centers are high-complexity intensive care units (ICUs); two have dedicated neurocritical care units led by intensivists and neurologists experienced in aneurysmal subarachnoid hemorrhage (aSAH) management.

SAMPLE SIZE

We performed a sample size calculation to ensure adequate precision in estimating the area under the Receiver Operating Characteristic Curve (AUROC) for Hunt and Hess mortality discrimination. Based on prior evidence, we assumed an AUROC of 0.80 and a mortality (event) proportion of 30%.(1) Under these assumptions, and targeting a 95% confidence interval (95%CI) width of 0.15 for the AUROC estimate, we required a minimum total sample size of 161 patients, corresponding to approximately 48 expected events. The sample size calculation was performed in Stata (version 16) using code for AUROC-precision-based sample size estimation, as proposed for predictive model validation.(2)

STATISTICAL ANALYSIS

Continuous variables were presented as mean and standard deviation (SD) or median and interquartile range (IQR), depending on distribution; categorical variables were presented as proportion with the absolute number.

To evaluate the predictive performance of the Hunt and Hess score, we first assessed discrimination and calibration. Discrimination measures the extent to which a model assigns a higher probability of an event to patients who will have it than to those who will not. Calibration reflects the extent to which a model correctly estimates the absolute risk. Poorly calibrated models will underestimate or overestimate the outcome of interest. Discrimination is quantified with a concordance (c) statistic that is identical to the AUROC curve for a binary outcome.(3) To evaluate the calibration, we compared the mortality predicted by the model with the observed proportion of mortality in each of the five possible scores of the Hunt and Hess. Secondly, we evaluate the model calibration with Cox's approach.(4,5) We applied this approach using the linear prediction (log odds) of each model as an independent variable and mortality as a binary outcome in the validation group. As calibration metrics, we estimated the calibration-in-the-large (CITL), the calibration slope, and the expected/observed ratio (E/O).

To describe neurological outcomes, we used the modified Rankin Scale (mRS) at hospital discharge and 6 months after discharge. We presented the proportion of patients with each mRS score, with 95%CI calculated using the Cooper-Person approach. Statistical analysis was performed with STATA v.16.

This is an open access article under the CC BY license (https://creativecommons.org/licenses/by/4.0/).

RESULTS

Between 2011 and 2022, 222 patients were admitted to the three hospitals with SAH. Of these, 46 were excluded due to traumatic SAH (Figure 1S). Finally, 175 patients were included, with an average age of 59 years (SD 14.4) and an average APACHE II score of 13 (SD 8.4). The most common Hunt and Hess grade was 2, observed in 36.6% (n = 64) of the patients. Regarding treatments, 60% (n = 106) received embolization, 32% (n = 56) underwent clipping, and the remaining patients did not receive aneurysm repair (demographic data in table 1). The overall mortality rate was 21% (n = 37).

For the primary outcome, we observed that the Hunt and Hess score showed good discrimination, with an AUROC of 0.74 (95%CI 0.66 - 0.82) for mortality prediction. However, in the calibration analysis, the Hunt and Hess score was found to over-predict mortality across all deciles (Figure 2S). Specifically, the CITL was -3.6, and the slope was 0.3. The negative CITL indicates that the predicted mortality was higher than the observed mortality. Additionally, a slope less than 1 suggests that the overprediction of mortality was more pronounced as the Hunt and Hess score increased (Figure 1). For example, for patients with a score of 1, an expected mortality rate of 10% was predicted, but no patients died. For those with a score of 5, an expected mortality rate of 99% was predicted, but only 46% of these patients died during hospitalization, indicating an overestimation of mortality.

To evaluate neurological outcomes, we divided the population into two groups: those with a good neurological grade (mRS 0 to 3) and those with a poor neurological grade (mRS 4 to 6). At discharge, the proportion of patients with mRS ≤ 3 was 49% (95%CI 41 - 56; n = 85). At 6 months, this proportion increased to 54% (95%CI 46, 62; N=81). Comparing mRS scores at discharge and 6 months, we observed improvement in neurological outcomes, particularly in patients with a Hunt and Hess score ≥ 4 at discharge. Specifically, 9% (n = 6) of the 69 patients with a Hunt and Hess score of 4 or 5 had an mRS ≤ 3 at discharge (Table 1S). Of these 69 patients, 60 were followed for 6 months, and 22% (n = 13) had an mRS ≤ 3 at the end of this period.

Finally, 1 year after aSAH, the survival rate was 72% (95%CI 64 - 78%), and at 5 years of follow-up, the survival rate was 70% (95%CI 61 - 76%).

REFERENCES

1. Rojas-Panta G, Reyes-Narro GF, Toro-Huamanchumo C, Choque-Velasquez J, Saal-Zapata G. Prognostic value of scales for aneurysmal subarachnoid hemorrhage: report of a reference center in Peru. Neurocirugia (Engl Ed). 2024;35(1):1-5.
2. Riley RD, Debray TP, Collins GS, Archer L, Ensor J, van Smeden M, et al. Minimum sample size for external validation of a clinical prediction model with a binary outcome. Stat Med. 2021;40(19):4230-51.
3. Huespe IA, Bisso IC, Roman ES, Prado E, Gemelli N, Sinner J, et al. Multicenter validation of Early Warning Scores for detection of clinical deterioration in COVID-19 hospitalized patients. Med Intensiva (Engl Ed). 2023;47(1):9-15.
4. Steyerberg EW, Vergouwe Y. Towards better clinical prediction models: seven steps for development and an ABCD for validation. Eur Heart J. 2014;35(29):1925-31.
5. Huespe IA, Lockhart C, Kashyap R, Palizas F Jr, Colombo M, Romero MD, et al. Evaluation of the discrimination and calibration of predictive scores of mortality in ECMO for patients with COVID-19. Artif Organs. 2023;47(6):1007-17.

Table 1S - Relation between Hunt and Hess score and modified Rankin Score at hospital discharge and 6 months after discharge

Hunt and Hess	mRS hospital discharge						
	0	1	2	3	4	5	6
1	9 (47)	5 (26)	1 (5)	4 (21)	0	0	0
2	24 (38)	17 (27)	5 (8)	3 (5)	6 (9)	4 (6)	5 (8)
3	3 (13)	2 (9)	9 (1)	6 (26)	7 (30)	1 (4)	4 (17)
4	0	2 (10)	1 (5)	0 (0)	3 (14)	7 (33)	8 (38)
5	0	1 (2)	1 (2)	1 (2)	11 (23)	14 (29)	20 (42)
**Hunt and Hess**	**mRS 6 months after patient discharge**						
	**0**	**1**	**2**	**3**	**4**	**5**	**6**
1	8 (57)	4 (29)	0	0	1 (7)	1 (7)	0
2	27 (48)	10 (18)	4 (7)	3 (5)	3 (5)	3 (5)	6 (11)
3	3 (15)	9 (30)	3 (15)	0 (0)	3 (15)	1 (5)	4 (20)
4	1 (7)	9 (7)	1 (7)	1 (7)	1 (7)	2 (13)	8 (53)
5	1 (2)	2 (4)	1 (2)	5 (11)	5 (11)	9 (20)	22 (49)

mRS - modified Rankin Scale. Results expressed as n (%).

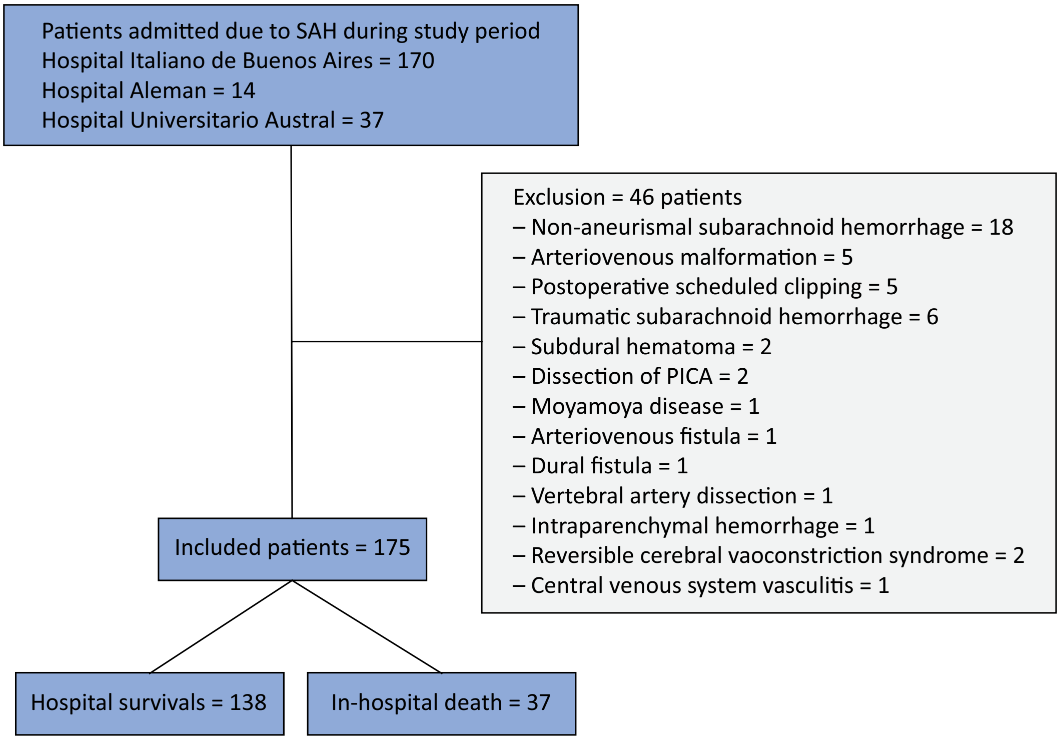

PICA - posterior inferior cerebellar artery.

Figure 1S - Flowchart.

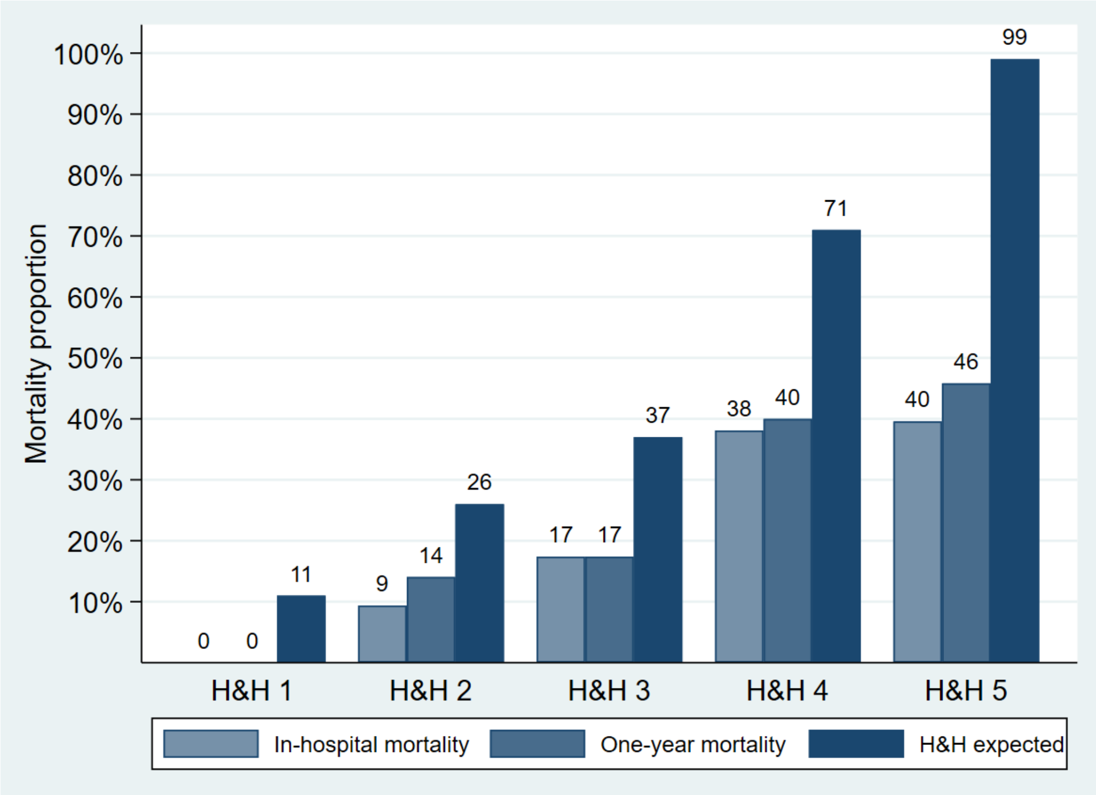

H&H - Hunt and Hess.

Figure 2S - Differences between the expected and observed mortality in the different scores of Hunt and Hess.
